# The Dentato-Rubro-Olivary Tract: Clinical Dimension of This Anatomical Pathway

**DOI:** 10.1155/2013/934386

**Published:** 2013-04-11

**Authors:** Fadil Khoyratty, Thomas Wilson

**Affiliations:** ENT Department, Leeds General Infirmary, Leeds, UK

## Abstract

Symptomatic palatal tremor is potentially the result of a lesion in the triangle of Guillain-Mollaret (1931) and is associated with hypertrophic olivary degeneration (HOD) which has characteristic MR findings. The triangle is defined by dentate efferents ascending through the superior cerebellar peduncle and crossing in the decussation of the brachium conjunctivum inferior to the red nucleus, to finaliy reach the inferior olivary nucleus (ION) via the central tegmental tract. The triangle is completed by ION decussating efferents terminating on the original dentate nucleus via the inferior cerebellar peduncle. We can demonstrate the anatomy of this anatomical triangle using a clinical case of palatal tremor presenting with bilateral subjective pulsatile tinnitus along with the pathognomonic MR findings previously described. The hyperintense T2 signal in these patients may be permanent, but the hypertrophied olive normally regresses after 4 years. The temporal relationship between the evolution of the histopathology and the development of the palatal tremor remains unknown as does the natural history of the tremor. Botox injection at the level of tensor and levator veli palatini insertion have been used to treat patients with disabling tremor synchronous tinnitus. A lesion involving the triangle can have a quite varied clinical expression.

## 1. Introduction

Palatal tremor (PT) was first described by Politzer in 1878 [1]. This rare condition exists in two variations, namely, an “essential” form where it is not attributable to a structural cause and a “symptomatic” form that is secondary to pathology involving the brainstem or cerebellum [2]. Symptomatic PT, also known as symptomatic palatal myoclonus is a clinically, anatomically, and pathologically well-defined movement disorder characterized by a stereotypic 1–3 Hz palatal contractions that commonly appear after an injury (e.g., haemorrhage, infarction, trauma, neoplasm, and demyelination) involving the Guillain-Mollaret triangle (G-Mt) also known as the dentato-rubro-olivary tract [2–4]. Dentatorubral tremor and ocular myoclonus are the other associated movement disorders. 

Symptomatic PT is correlated with hypertrophic olivary degeneration (HOD), a rare form of transsynaptic degeneration causing hypertrophy of the inferior olives as opposed to atrophy observed in other parts of the central nervous system [2, 4]. Oppenheim was the first to provide postmortem evidence of inferior olivary enlargement [5]. Antemortem visualization of HOD using MRI has helped to better characterize the causative neuropathologies and associate temporal evolution of the inferior olivary nuclei (ION) [3].

## 2. Case Report

A 60-year-old gentleman reports spontaneous onset subjective pulsatile tinnitus involving both his ears, of 2 years duration that gets worse when he is stressed and lies down. He denies any gross hearing loss and has no relevant past medical history. 

**Figure 1:**
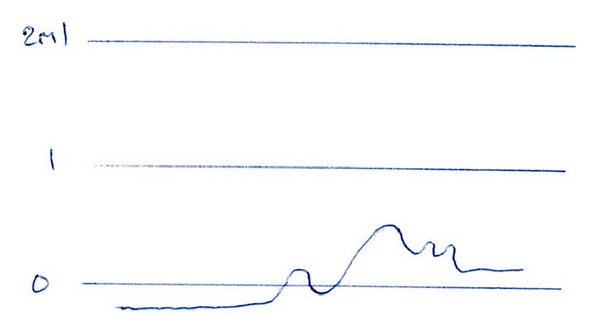
Variations in ear canal volume due to pulsating tympanic membrane.

Clinical and otoscopic examinations were essentially normal except for a palatal myoclonus which was at the same frequency as his tinnitus. Pure tone audiometry results were consistent with physiological hearing loss, and an attempted long time based tympanometry was suggestive of tympanic pulsation (Figure 1).

**Figure 2:**
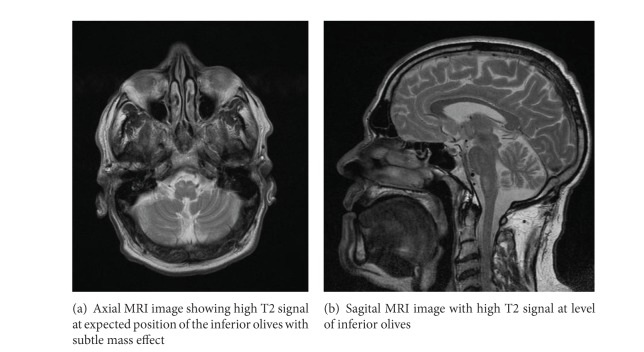
Isolated bilateral ION enlargement without accompanying brainstem or cerebellar pathology.

His serum biochemistry was normal as was his temporal bone imaging (CT scan). Primary brain imaging (MRA) showed a high bilateral T2 signal with subtle mass effect on the expected position of the olivary nuclei, a finding that is consistent with HOD (Figures 2(a) and 2(b)). Radiological examination of the remainder of the brain and midline structures was normal.

Following failure of pharmacotherapy (oral carbamazepine), a trial of local anaesthetic injection in clinic was aimed to achieve temporary paralysis of the muscles of the soft palate. This was followed by bilateral palatal botox injection at the level of the tensor and levator veli palatini insertion resulting in significantly improved tremor synchronous tinnitus.

## 3. Discussion

 Knowledge of the components of the G-Mt is essential for understanding how lesions affecting the triangle can influence the inferior olivary nuclei. The triangle is composed of the contralateral dentate nucleus, ipsilateral red nucleus, and the ipsilateral inferior olivary nucleus.

The red nucleus receives most of its fibres from the dentate, but there are also contributions from the emboliform and globose nuclei. Efferents from the dentate nucleus ascend through the superior cerebellar peduncle or brachium conjunctivum and decussate in the caudal midbrain to finally reach the contralateral red nucleus. The rostral third of the red nucleus (parvicellular part) is the end point of the dentatorubral pathway where they have asymmetrical synapses. Fibers from the parvicellular part of the red nucleus descend ipsilaterally via the central tegmental tract to reach the dorsal lamella of the principal inferior olivary nucleus. The triangle is completed by decussating fibers originating from the inferior olivary nucleus, forming the largest component of the inferior cerebellar peduncle (corpus restiform) and terminating on the original dentate nucleus [3]. This is a bidirectional pathway, a coupled system likely to be of a feedback function, because there are also projections from the dentate nuclei to the contralateral caudal inferior olivary nucleus. The inferior olive has an intrinsic slow, rhythmic, and spontaneous activity [6].

 Despite being considered to be a complete functional triangle, symptomatic PT and HOD only occur with lesions involving the first two limbs of the triangle and not with interruption of the olivodentate fibres (associated with cerebellar atrophy), since it is olivary deafferentation that is thought to trigger the hypertrophic degenerative changes [3]. 

 HOD is usually a unilateral phenomenon that is ipsilateral to the lesion if the lesion is found in the brainstem and contralateral to the lesion if the lesion is found in the cerebellum. There have been however reports of unilateral lesions causing bilateral HOD. Bilateral HOD secondary to a right cerebellar artery infarct that occurred during a craniotomy has been reported [7]. The reason for this remains unclear. A midline lesion at the level of the brachium conjunctivum will result in bilateral HOD as the ducassating fibers of the right and left dentate olivary tracts are likely to be involved.

 Our reported case, despite having MRI evidence of bilateral HOD, bears no underlying explainable pathology at the brainstem or cerebellar level. This is to the best of our knowledge the first reported case of adult onset symptomatic PT secondary to idiopathic bilateral HOD. A recent paediatric series has reported similar MRI findings in a patient not displaying an HOD related tremor [8]. This supports the fact that, although virtually all patients who develop palatal myoclonus after a brain insult are likely to have HOD, not all patients with HOD have palatal myoclonus [9].

 The evolving microscopic pathological changes seen in HOD have been well described using postmortem studies. Evidence of neuronal hypertrophy is first demonstrated at 3 weeks, with a peak in neuronal and glial growth observed at 8-9 months. Pseudohypertrophy (neuronal dissolution and gemistocytic astrocytes) follows that stage, ultimately leading to olivary atrophy [10, 11]. The sequential MR changes in HOD have similarly been described. In the first 6 months following the insult, there is an increased signal on T2 with normal looking inferior olives. The second stage lasts for about 3 to 4 years demonstrating both an increased signal with evidence of olivary hypertrophy. The third stage begins at the time when the hypertrophy resolves with a persistently high T2 signal [3]. These MR findings do correlate with the histopathologic changes of the ION.

 A recent postmortem study has suggested that alterations of the structures of the G-Mt can illustrate an important role in the pathogenesis of sudden unexplained fetal and infant death. The pathologies observed in these cases were hypoplasia/hyperplasia of the dentate, ION, and red nucleus with high neuronal c-fos expression (marker of neuronal activity). These changes have been thought to be due to low oxygen tension in the fetal circulation with maternal cigarette smoking, a contributing factor [12].

## 4. Conclusion

 Study of this neuroanatomical pathway has evolved from the first descriptions of inferior olivary enlargement, through Guillain and Mollaret's subsequent work describing the functional triangle in 1931 [13]. More recent antemortem MR imaging has highlighted the clinical significance of the individual tracts of the G-Mt, and this correlates with immunohistochemical [12] and ultrastructural electron microscopic [8] characterisation of the individual nuclei of the triangle. 

The clinical manifestation offered by alterations in the G-Mt is very much varied with no unifying theory able to link the pathological changes with resulting signs. There do however appear to be some close associations with movement disorders such as palatal tremor and cerebellar atrophy with a final, speculative association to unexplained perinatal death.

## Supplementary Material

Clinical Video Comments: palatal myoclonus having similar frequency as the pulsatile tinnitus reported by patient.
